# Triplane Fracture of the Proximal Tibia: A Case Report and Literature Review

**DOI:** 10.1155/2017/6490728

**Published:** 2017-09-18

**Authors:** Jason Strelzow, Alexander Aarvold, Christopher W. Reilly

**Affiliations:** BC Children's Hospital, University of British Columbia, Vancouver, BC, Canada

## Abstract

**Background:**

The triplane fracture, a unique transitional physeal injury, is classically described in the distal tibia. A small number of additional anatomic locations are documented in the orthopaedic literature.

**Methods:**

Available literature surrounding triplane fractures was reviewed. We describe a rare case of a proximal tibial triplane fracture in a thirteen-year-old girl, suffered during a skiing accident.

**Results:**

Using arthroscopically assisted percutaneous reduction techniques an anatomic reduction was achieved.

**Conclusion:**

We outline the surgical and postoperative techniques for management of this unique injury.

## 1. Introduction

Physeal fractures of the proximal tibia account for 1% to 3% of all physeal injuries [1–3]. Salter-Harris types I to V occur, from both indirect and direct mechanisms of injury [2]. These injuries may be associated with ligamentous injuries, vascular injuries, and compartment syndrome due to the close proximity of neurovascular structures and often high energy mechanism [2]. Within the available literature, triplane fractures of the proximal tibia have only been reported in eight cases [4–10]. These occurred due to motorcycle, bicycle, and sledding accidents as well as one sustained from being jumped on by an older sibling [6–8]. Two cases sustained a concomitant tibial spine avulsion fracture [6, 10] and another contralateral femoral shaft fracture [9].

Triplane fractures are classically described in the distal tibia in the 12–15-year-old age group [11, 12]. These injuries typically occur as a result of external rotation of the foot in the presence of the asymmetric physiologic epiphysiodesis characteristic for the distal tibial physis. These injury patterns elude simple classification based on the Salter-Harris classification of physeal injuries. Conventional criterion requires three-plane involvement occurring in the sagittal, coronal, and axial planes and can be two, three, or four parts [12]. We describe an extremely rare case of a triplane fracture of the proximal tibia, together with imaging and surgical strategy.

## 2. Case Report

**Figure 1:**
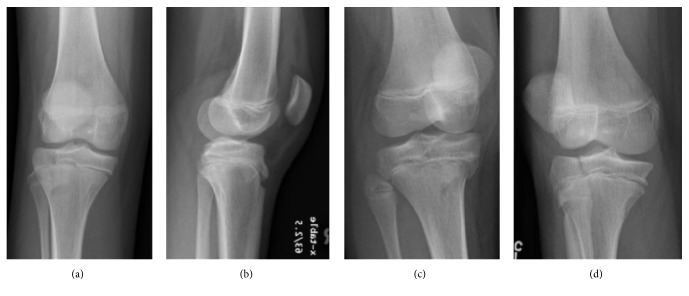
Anteroposterior (a), lateral (b), and oblique (c and d) radiographs of the patient's right knee.

**Figure 2:**
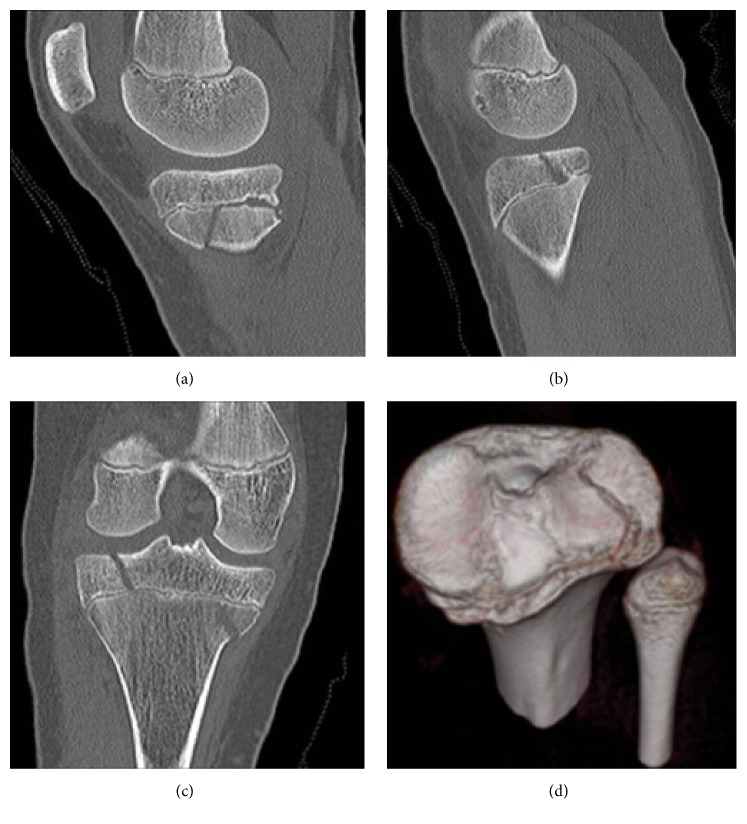
Sagittal, coronal, and 3D reformatted CT images of the right knee demonstrating the coronal fracture medially in the metaphysis (a), laterally in the epiphysis (b), sagittal fracture lines (c), and the rotational component in the axial plane (d).

A 13-year-old female hit a tree while skiing, sustaining a direct blow combined with a twisting injury to her right knee. This was an isolated injury and her management was escalated to our institution, a tertiary referral children's' hospital. She had an acute tense haemarthrosis, painful range of motion, and no neurovascular deficit nor signs of compartment syndrome. Radiographic findings of a fracture involving the tibial plateau (Figure 1) were clarified on computed tomography, demonstrating coronal (Figures 2(a) and 2(b)), sagittal (Figure 2(c)), and axial (Figures 2(c) and 2(d)) components to her injury. Three-dimensional (3D) reformats illustrated the two-fragment triplane fracture (coronal at the level of the epiphysis, sagittal at the metaphysis, and axial at the physis) and was notable for the internally rotated displacement of the tibial metaphysis relative to the proximal physis (Figure 2(d)).

## 3. Operative Technique

**Figure 3:**
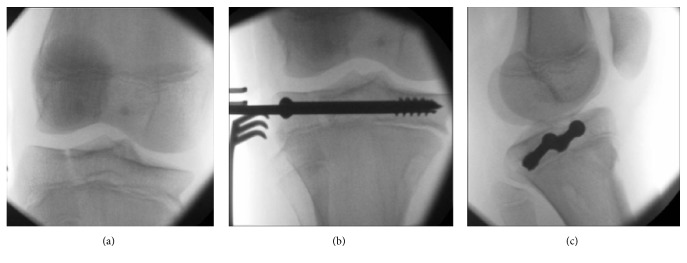
Intraoperative images of closed reduction (a), percutaneous fixation (b), and compression (c) of the right knee.

**Figure 4:**
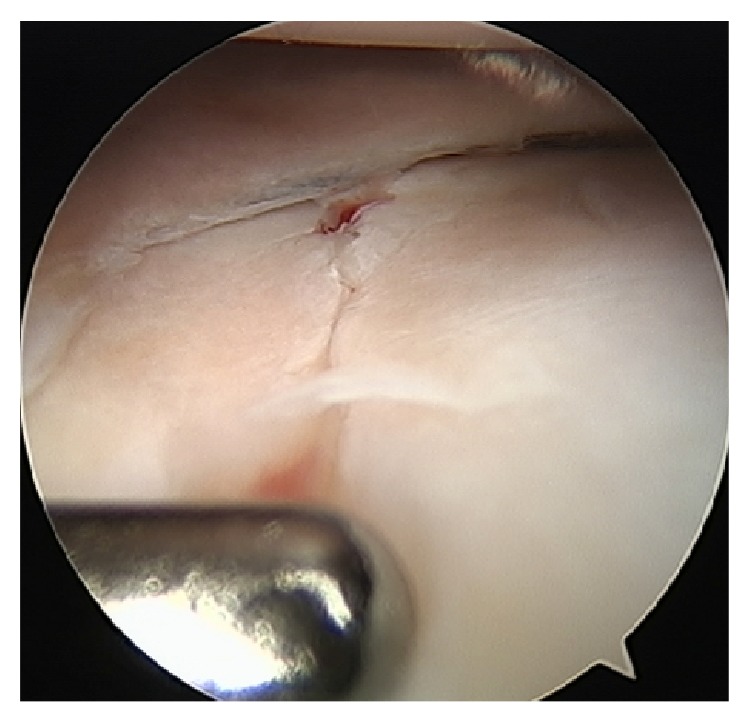
Arthroscopic confirmation of the tibial plateau joint line reduction with a 3.4 mm hook probe (Arthrex, FL, USA) in the foreground.

Under general anaesthetic, the patient was positioned supine with a tourniquet on the thigh. Under fluoroscopic guidance, a closed reduction was performed by traction, extension, and external rotation of the tibial shaft relative to the proximal physis (Figure 3(a)). The articular surface was compressed and held using partially threaded 7.3 mm cannulated screws in the epiphysis (Figures 3(b) and 3(c)). The large screw thread size was chosen to maximize compression of the fracture. A diagnostic arthroscopy was performed, confirming the articular surface reduction (Figure 4) and excluding concomitant injury to the ACL, tibial spine, and menisci and the presence of osteochondral fragments.

## 4. Postoperative Course

The patient was immobilized in a long leg cast in partial flexion, non-weight-bearing, for four weeks. Graded increase in range of motion and weight bearing was introduced over six weeks, supported by a hinged knee brace and physiotherapy. Screw removal is planned for 6–12 months postoperatively.

## 5. Discussion

The triplane fracture occurs due to the asymmetrical closure of the distal tibial physis, from central to anteromedial to posteromedial, and finishing with closure of the lateral margin of the physis [13]. The proximal tibial physis has a more symmetrical closure, which may explain the rarity of a triplane fracture in this location. However, the literature is somewhat unclear regarding a defined pattern, unlike the distal tibia. Haines described the physical closure pattern starting initially from the anteromedial tibia and proceeding posterolaterally but other studies suggest more peripheral to posterior or central to peripheral patterns [13–16]. Further factors that may contribute to the rarity of this injury are the insertion of the collateral knee ligaments into the tibial metaphysis (resulting in stresses bypassing the epiphysis, unlike in the ankle) and tibial spine and tibial tubercle avulsions off-loading the strain and relatively protecting the physis.

3D imaging is particularly helpful in Salter-Harris type III and IV fractures and triplane injuries due to the articular surface involvement and fracture orientation [1, 4, 5, 10, 17]. CT imaging was critical in this case to map the morphology of the fracture, allowing surgical planning for the reduction sequence. Thus percutaneous screw placement was possible and the zone of injury was protected. The primary goals of treatment are anatomic reduction of the articular fragments with avoidance of further injury to the growth plate. Arthroscopy was valuable to directly visualize the reduction of the articular surface (Figure 4) and subsequently assess internal knee structures which are so hard to assess clinically in the acutely painful, swollen knee.

This case of a rare triplane fracture of the proximal tibia illustrates the often complex morphology of these injuries and the minimally invasive techniques that can be used to manage the combined articular and physeal injuries. The importance of both 3D imaging and arthroscopic assessment has been demonstrated, providing further insight into this unusual fracture pattern.
